# Knowledge Translation of the PERC Rule for Suspected Pulmonary Embolism: A Blueprint for Reducing the Number of CT Pulmonary Angiograms

**DOI:** 10.5811/westjem.2017.7.34581

**Published:** 2017-09-18

**Authors:** Michael J. Drescher, Jeremy Fried, Ryan Brass, Amanda Medoro, Timothy Murphy, João Delgado

**Affiliations:** * Hartford Hospital, Department of Emergency Medicine, Hartford, Connecticut; † University of Connecticut School of Medicine, Department of Emergency Medicine, Farmington, Connecticut; ‡ Rabin Medical Center, Department of Emergency Medicine, Petah Tikvah, Israel; § Charlotte Hungerford Hospital, Department of Emergency Medicine, Torrington, Connecticut; ¶ Rutland Regional Medical Center, Department of Emergency Medicine, Rutland, Vermont. University of New Mexico, Department of Emergency Medicine, Albuquerque, New Mexico; || University of Cincinnati College of Medicine, Department of Emergency Medicine, Cincinnati, Ohio

## Abstract

**Introduction:**

Computerized decision support decreases the number of computed tomography pulmonary angiograms (CTPA) for pulmonary embolism (PE) ordered in emergency departments, but it is not always well accepted by emergency physicians. We studied a department-endorsed, evidence-based clinical protocol that included the PE rule-out criteria (PERC) rule, multi-modal education using principles of knowledge translation (KT), and clinical decision support embedded in our order entry system, to decrease the number of unnecessary CTPA ordered.

**Methods:**

We performed a historically controlled observational before-after study for one year pre- and post-implementation of a departmentally-endorsed protocol. We included patients > 18 in whom providers suspected PE and who did not have a contraindication to CTPA. Providers entered clinical information into a diagnostic pathway via computerized order entry. Prior to protocol implementation, we provided education to ordering providers. The primary outcome measure was the number of CTPA ordered per 1,000 visits one year before vs. after implementation.

**Results:**

CTPA declined from 1,033 scans for 98,028 annual visits (10.53 per 1,000 patient visits (95% CI [9.9–11.2]) to 892 scans for 101,172 annual visits (8.81 per 1,000 patient visits (95% CI [8.3–9.4]) p<0.001. The absolute reduction in PACT ordered was 1.72 per 1,000 visits (a 16% reduction). Patient characteristics were similar for both periods.

**Conclusion:**

Knowledge translation clinical decision support using the PERC rule significantly reduced the number of CTPA ordered.

## INTRODUCTION

### Background

In recent years the pursuit of the diagnosis of pulmonary embolism (PE) has been the focus of much discussion in the medical literature. PE is common, difficult to diagnose, and potentially lethal if missed.^1^ Computed tomography pulmonary angiogram (CTPA) is currently the preferred test to diagnose PE. However, excessive testing in the pursuit of the diagnosis of PE has long been an area of concern.^2^

To enable clinicians to confidently rule out PE while reducing the number of unnecessary CTPAs, several clinical rules have been developed and validated. These include the Wells criteria and the Pulmonary Embolism Rule-Out Criteria (PERC).^3^,^4^ A low-probability Wells score, along with a negative D-dimer^5^ or a negative PERC score, can rule out a PE with a high enough degree of certainty that the number of patients who would benefit from further testing to increase that certainty would be less than the number harmed by the side effects of the testing itself and the harmful consequences of false-positive results, including needless anticoagulation.^6^ Despite clear evidence that the use of validated clinical rules can effectively be used to rule out the diagnosis of PE, anecdotally they are not universally or even commonly applied.

Previous studies have described the use of computerized clinical decision support (CDS) rules whereby clinicians are reminded of relevant clinical rules during the ordering process. However, the effectiveness of this approach has been inconsistent. Raja et al. reported success in reducing the number of CTPA ordered and increase in yield, whereas Drescher et al. reported that CDS was unsuccessful in reducing the number of CTPA ordered and was not accepted by the clinicians ordering the tests.^7^,^8^ In the former study, some limited physician education was done prior to implementing the CDS, whereas in the latter none is described. In addition, these studies used CDS based on Wells criteria for risk assessment of PE along with the use of D-dimer testing. PERC is an established clinical rule by which low-risk patients can be safely ruled out for the diagnosis of PE without the use of any ancillary testing. Our CDS was designed to incorporate this validated clinical rule along with Wells criteria and D-dimer testing as needed. To our knowledge, this is the first reported incorporation of PERC into a computerized CDS system.

Knowledge translation (KT) is a field of endeavor defined by the Canadian Institutes of Health as “a dynamic and iterative process that includes the synthesis, dissemination, exchange and ethically sound application of knowledge to improve health, provide more effective health services and products, and strengthen the health care system.”^9^ This definition recognizes the fact that knowledge creation and dissemination is not sufficient to affect clinical care and decision-making. We designed an explicit KT-based educational process to accompany the introduction of our CDS tool. We hypothesized that the combination of a CDS tool, a targeted education effort, and regular feedback to providers on their utilization rates would reduce the number of unnecessary CTPA ordered.

### Importance

Excessive use of CTPA in the emergency department (ED) is associated with risks and costs to patients and systems of care. Although the increased risk of cancer to an individual patient from a single CTPA is low, the stochastic effects of ionizing radiation from CTs increase the population incidence of neoplastic disease.^10^ In addition, the use of CTPA involves contrast medium, which can be nephrotoxic and lead to allergic reactions. Severe, life-threatening reactions including anaphylaxis occur in 0.1% of people receiving contrast media.^11^ Recently, an increase in major adverse advents associated with CT contrast-induced nephropathy has been reported.^12^ Testing for PE also carries implications for cost, throughput, patient satisfaction and resource allocation, as well as the risks of long-term anticoagulation in patients with false-positive or clinically insignificant positive findings.

**Population Health Research Capsule**

What do we already know about this issue

Despite clear evidence that validated clinical decision rules can be used to rule out pulmonary embolism (PE), they are not universally or even commonly applied.

What was the research question?

We hypothesized that a clinical decision support tool, education effort, and regular fee dback to providers would reduce the number of unnecessary computed tomography pulmonary angiograms (CTPA) ordered.

What was the major finding of the study?

The number of CTPA per 1,000 patients decreased from 10.53 to 8.81, a 16% relative reduction.

How does this improve population health?

This study addressed indiscriminate CTPA testing for PE in pursuit of a diagnosis, which could result in over-diagnosis, harm patients and divert resources from other health care needs.

### Goals of this Investigation

We hypothesized that a coordinated KT effort involving the adoption of a department-endorsed, evidence-based clinical protocol, a multi-modal educational program, and CDS embedded in our computerized order entry system, would lead to a decrease in the number of CTPA ordered.

## METHODS

### Study Design and Setting

This was an observational before-after study in which data were collected prospectively over 12 months starting on October 15, 2012, and compared to the previous 12 months. The study took place in the ED of an urban tertiary referral center with a large emergency medicine (EM) residency program and with an annual census of 100,000 visits.

### Patient Population

Our study population included all non-pregnant patients over the age of 18 in whom the providers suspected a diagnosis of PE and who did not have a contraindication to CTPA, including renal insufficiency and allergy to contrast material.

### Intervention Protocol

A departmental protocol was instituted so that to obtain a CTPA for possible PE, providers were required to enter clinical information into a departmentally-approved diagnostic pathway based on recommendations of the American College of Emergency Medicine guidelines^1^ regarding the evaluation of adult patients in the ED for PE. This diagnostic pathway was embedded into the computerized order entry system (Figure). The protocol required that a Wells score be calculated for all patients suspected of having a PE.

Low-risk patients then had their PERC score calculated. Patients with a negative PERC score were considered ruled out for PE with no further testing. Those patients deemed low risk by Wells score who did not meet the PERC criteria had D-dimer testing. Intermediate-risk patients had D-dimer testing, and high-risk patients had a CTPA ordered. Those patients who had negative D-dimer results were considered ruled out for PE and had no further testing. The computerized algorithm displayed the protocol pathway and guided providers according to their responses. However, to allow the ultimate decision to remain with the clinician at the bedside, providers were able to override the protocol and order a CTPA even if not indicated by the protocol. In these cases we sent an email to the ordering provider inquiring as to their reasoning for ordering CTPA despite the negative evaluation. We did not require or tally responses to these emails.

### Knowledge Translation Implementation

Prior to implementation of the protocol, education was provided to ordering providers (attending and resident physicians, physician assistants, and advanced practice registered nurses) in the ED about the clinical rules and how to complete the ordering process. Discussion was invited at departmental meetings. An authority in the field of KT and the rational diagnosis of PE was invited to give a grand rounds talk on the topic. Subsequent to protocol implementation, providers who ordered CTPA outside the protocol parameters were reminded of the protocol by email and queried as to the reason for the violation. In addition, a quarterly utilization report was generated, distributed by email, and displayed during monthly staff meetings so that attending physicians could see their utilization of CTPA relative to that of their peers. Included in the process were 17 clinical EM faculty and 36 EM residents.

### Data Collection

We identified all CTPAs performed during the study period through a query of the clinical system (Allscripts ED, Chicago, IL). Trained abstractors reviewed each record and recorded basic demographics, D-dimer and CTPA results, whether the CT was ordered according the departmental guidelines, and in cases of non-compliance, which specific item(s) were not adhered to. Educational sessions were held for the research assistants prior to the start of data collection, and one of the investigators (JF) audited 20% of all the charts to ensure data accuracy. Discrepancies were reconciled by referring to the source documentation. Data collection for the baseline period was performed retrospectively while data collection following implementation of the protocol was performed prospectively. We collected and managed study data using REDCap^13^ electronic data capture tools hosted by the Hartford HealthCare Research Program. The study was approved by the hospital institutional review board.

### Outcome Measures

The primary outcome measure was a reduction in the number of CTPA ordered per 1,000 patient visits in the year after the guideline was implemented compared to the year before. Secondary outcome measures were yield of CTPA ordered (percentage of positive tests) and compliance with the CTPA ordering guideline.

### Analysis

The primary analyses for this study were comparison of CTPAs per 1,000 patient visits and the positive yield for PE between the periods of time before and after the implementation of the guideline and training. We calculated the rate of CTs performed for this purpose as the number of CTs performed per 1,000 ED visits before and after protocol implementation. Descriptive statistics for the cohort are expressed as means with standard deviations (SD) and proportions. Inferential statistics are expressed as point estimates with 95% confidence intervals (CI). We analyzed differences in means and proportions with *t* tests or chi-square analysis, as appropriate.

We conducted additional analyses for the post-implementation period only, due to availability of data. These focused on whether the guidelines were followed or a violation occurred, specifically whether a D-dimer was ordered and positive, and whether Wells or PERC criteria were followed. We created comparison groups based on the findings for these violations and compared them for the final diagnostic accuracy, again using chi-square tests of proportion. We performed all analyses with MedCalc version 13.1.2 (MedCalc Software, Ostend, Belgium) or with SPSS version 21 (SPSS, Inc., Chicago, IL).

## RESULTS

There were no significant differences in the age or gender of the patients receiving CTPA in the periods before and after the interventional protocol was adopted. The average age was 59 in the period before and 59.4 in the period after. The proportion of males in the period before was 40.1% and 42.9% in the period after.

The total number of CTPAs declined from 1,033 scans for 98,028 annual visits to 892 scans for 101,172 annual visits. The number of CTPAs per 1,000 patient visits decreased from 10.53 (95% CI [9.9 to 11.2]) for the year before the guideline was enacted to 8.81 (95% CI [8.3 to 9.4]) for the year after (absolute difference 1.72, p<0.001). This difference represents a 16% reduction in CTPA utilization. The secondary outcome measure of PE yield showed no significant change, from 15.9% positive CTPA for PE in the year prior to 15.2% positive for PE in the year after (p=NS). The protocol was followed 66% of the time. When the protocol was followed (n=589) the positive PE yield was 18.4%; when it was not (n=303), the yield was 9.2% (p < 0.001). Types of protocol violations were subdivided, with multiple violations occurring in some cases (Table).

D-dimer was ordered in 34.4% of cases before and 65.7% of cases after protocol implementation (p = 0.001). For those patients who had a D-dimer ordered there was a significant increase in the proportion of elevated (>250 ng/ml not age adjusted) D-dimers from 91.8% before to 95.9% after (p = 0.009).

## DISCUSSION

The diagnosis of PE in the ED is of paramount importance. It is a disease with a frightening combination of being common, difficult to diagnose and potentially lethal if missed. On the other hand, indiscriminate pursuit of the diagnosis using CTPA harms patients and wastes resources. Diagnosis of clinically inconsequential PE or false-positive CTPA may also lead to unnecessary anticoagulation and increased ordering of subsequent CTPA, as well as increased and needless patient anxiety.

Our study was ultimately an attempt to address the challenges of unnecessary testing and overdiagnosis in the context of diagnosing PE. Five of the medical specialty societies participating in the *Choosing Wisely* campaign, including the American College of Emergency Physicians, have listed avoiding unnecessary imaging for PE among their main recommendations.^15^ Similarly, avoiding PE overdiagnosis is included in the *British Medical Journal*’s *Too Much Medicine* campaign.^16^

The term overdiagnosis can be used in a broad sense to refer to several related concepts: overdetection of disease, overmedicalization of common human conditions, overutilization of resources, and overtreatment.^17^ As diagnostic modalities have improved and illness definitions expanded, overdiagnosis has become a global problem for modern medical practice. The adverse consequences of overdiagnosis have been described for conditions as diverse as asthma, attention deficit hyperactivity disorder, breast cancer, hypertension, and PE.

Radiation and cost concerns aside, applying a highly sensitive test (CTPA) to a population with low PE prevalence leads to two distinct problems: diagnosing small PEs that will not harm the patient (overdetection) and diagnosing PEs that are not there (false positives), decreasing the specificity of the test. Either case will lead to unnecessary anticoagulation with all its attendant risks and costs. Effective KT can help clinicians avoid finding these PEs that should not be found, and in the process save money while providing the safest care for patients.

Consequently, much research has been devoted to developing a risk-stratification strategy that has been shown to safely rule out the diagnosis of PE in a significant subgroup of patients without the use of CTPA. To maximize the benefits to patients of such an evidence-based strategy it needs to be implemented in a systematic way. The principles of KT (also known as dissemination and implementation) provide a framework for doing this. Different formal frameworks have been described. There are differences between the frameworks, but they all retain some common characteristics: an evidence-based intervention with demonstrated effectiveness, guided implementation and innovation, evaluation, sustainability, and stakeholder input.^18^

The protocol has been shown to be sustainable in our department as it has been implemented and retained as the standard method for ordering CTPA to rule out PE. Our protocol followed the principles of KT in bringing evidence-based knowledge to bear and successfully affecting patient care at the departmental level. We followed a path of provider education, getting the buy-in of decision-makers, establishing a department standard and reiterating the expectations through a feedback loop.

The pursuit of the diagnosis of PE is well suited to this pathway. The stakes are high both for patient outcomes and resource utilization, The evidence for influencing practice is well established in the literature, and the clinical rules are well known and lend themselves to incorporation into computerized order entry algorithms. The need for translation of this knowledge into practice is demonstrated by the seminar workshop given yearly by the largest EM conference in the world entitled “Stop the Madness I: Reducing Unnecessary Radiation in Suspected Pulmonary Embolism.” 19 Nonetheless, to date we are not aware of a published study showing the feasibility and results of implementation of KT of PERC in the diagnosis of PE.

Importantly, we did not measure the number of missed PE in adopting our protocol. The safety of the protocol, including the use of the PERC rule as part of the algorithm, has been previously established in the literature and endorsed in guidelines by major medical professional organizations.^20^ We therefore did not see the need to re-evaluate the safety and accuracy profiles of the components of our intervention; rather, we set out to assess the effectiveness of implementation in practice, as an established, evidence-based pathway.

The present study demonstrated a significant decrease in utilization of CTPA when the principles of KT, in which a dynamic and iterative process that includes the synthesis, dissemination, exchange and ethically-sound application of knowledge, were applied to the diagnosis of PE using a novel CDS, which included PERC criteria to risk stratify along with Wells criteria for PE and D-dimer testing.

## LIMITATIONS

The main limitations of this study are due to its observational design. One would expect that with the decrease in utilization observed in our study, there would be a concomitant increase in yield, if the prevalence of PE were unchanged. We did not find an overall increase in yield despite a decrease in utilization. This raises the question of whether the protocol resulted in more missed PEs along with the decrease in utilization of CTPA. However, we did find an increase in yield when the protocol was followed and a significant decrease in yield when the protocol was violated. This is consistent with the evidence on which this study is based, showing that patients who are risk stratified by clinical rules prior to decision-making on whether to order CTPA will have fewer negative tests than those who are not. In addition, it is possible that in using the protocol, patients with clinically insignificant PE were scanned at a lower rate. A higher rate of small, clinically insignificant PE would have increased the total yield in the pre-protocol group reducing the change in yield seen after protocol implementation. We did not tally protocol violations by provider. It is possible that if providers who were risk averse were disproportionate violators of protocol, this would further lower positive yield in this group.

There is a necessary threshold for considering PE in the differential diagnosis for a given patient. It is possible that in the pre-protocol period, where there was no structured risk stratification, that the threshold for consideration of PE was higher than after protocol implementation. The threshold for considering patients for entry into the protocol could not be known. It is possible that a larger group of patients at low risk for PE were included than otherwise would have been, thus decreasing the diagnostic yield in the post-implementation period.

It is possible that a secular trend occurred over the time our intervention was implemented and that, for example, patients during the intervention period presented with fewer risk factors and signs of PE than during the pre-intervention period, which may have led to fewer scans ordered even without our implementation of the clinical protocol. We have no reason to suspect this or any other secular trend. We did not follow the clinical course of our patients to determine if there were any missed PEs before or after the protocol implementation.

An additional potential criticism of our study is that because we used multiple interventions (grand rounds, faculty meeting discussions, individual provider audit and feedback, electronic medical records,) we were not able to delineate the value of these interventions individually on our results. This was deemed impractical for two reasons. First, our main goal was to reduce the number of CTPAs ordered, and we pursued multiple avenues to achieve that mission. Second, individual clinicians are not influenced by differing interventions uniformly. While some may be more swayed to change behavior based on updated clinical evidence presented at grand rounds, others may be more influenced by the change in ordering procedure found in the EMR; and the individuals themselves often have little insight into their own thought processes.^14^ So while we did not explicitly study the level of acceptance by providers of the protocol after implementation, we do not view this as a true limitation of the study since the true imCTPA on patients is in the outcome measured, that is rate of CTPA ordered, by the protocol implementation as a whole. We did not assess the impact of the protocol for increase or decrease in compliance over time.

## CONCLUSION

Application of the principles of knowledge translation to an evidence-based, ED-mandated ordering process that includes the PERC rule significantly reduced the number of CTPAs ordered.

## Figures and Tables

**Figure:**
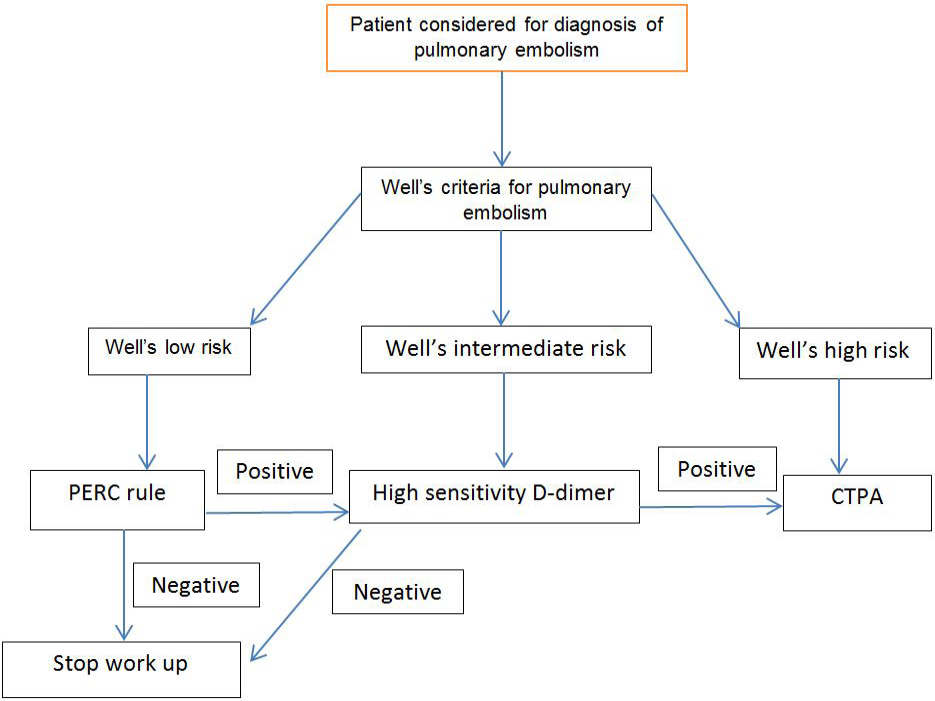
Diagnostic pathway embedded into computerized order entry system.

**Table:** Type of protocol violation regarding lack of adherence to clinical decision support pathway for diagnosis of pulmonary embolism.

Type of protocol violation	Percentage of cases
D-dimer not done or CTPA done despite negative result	15.1
Wells Score not calculated	18.2
Pulmonary embolism ruleout criteria not assessed	5.5
Other violation	3.5

*CTPA*, computed tomography pulmonary angiograms.
